# Psychotic experiences in university students: prevalence, correlates and association with non-specific psychological distress

**DOI:** 10.1192/j.eurpsy.2024.459

**Published:** 2024-08-27

**Authors:** N. Werbeloff, N. Sobol

**Affiliations:** School of Social Work, Bar Ilan University, Ramat Gan, Israel

## Abstract

**Introduction:**

Subclinical psychotic experiences (PEs) are far more prevalent than psychotic disorders, with an estimated prevalence of 7.2% (Linscott & Van Os. Psychol Med 2013;43(6) 1133-1149). PEs are particularly prevalent in late adolescence and young adulthood, when obtaining academic education is one of the main developmental tasks. University students are at the peak age of onset of mental disorders, and often experience high levels of social and academic stress that may contribute to the onset of psychopathology. Hence, estimating the prevalence and correlates of PEs among university students is particularly important.

**Objectives:**

To estimate the prevalence of PEs in a sample of Israeli students; assess whether rates of PEs differ by selected sociodemographic characteristics; and examine the association between PEs and non-specific psychological distress.

**Methods:**

150 students from universities and colleges in Israel participated in a cross-sectional online survey. All students were over the age of 18 and were not diagnosed with psychotic disorders. Participants completed self-report questionnaires, including the Prodromal Questionnaire - Brief Version (PQ-B), Kessler Psychological Distress Scale (K10) and sociodemographic details. The PQ-B yields a score for the total number of items endorsed (range 0–21), and a total distress score (range 0–105). A cutoff of ≥8 distressing symptoms was used to identify participants at high-risk for psychosis.

**Results:**

21 participants (14.0%) reported 8 or more distressing PEs. PEs were more common in males and among those with a psychiatric illness (Table 1). PEs were not associated with marital status, religiosity, or immigrant status. While a greater number of PEs was positively associated with non-specific psychological distress (r=0.589, p<.001), there was no association between distress caused by PEs and non-specific psychological distress (r=0.145, NS).

**Table 1.** Sociodemographic characteristics by group

		PEs-	PEs+	X^2^, p
Sex	M	29.5%	52.4%	4.32, .038
	F	70.5%	47.6%	
Marital Status	Married	17.1%	23.8%	0.56, NS
	Unmarried	82.9%	76.2%	
Immigrant	No	89.9%	85.7%	0.34, NS
	Yes	10.1%	14.3%	
Religiosity	Secular	74.4%	57.1%	2.67, NS
	Other	25.6%	42.9%	
Psychiatric illness	No	87.6%	61.9%	8.87, .003
	Yes	12.4%	38.1%

**Conclusions:**

The findings confirm that self-reported PEs are much more prevalent than clinically diagnosed psychotic disorders, particularly among young adults. As PEs were found to be associated with non-specific psychological distress, and as they are known forerunners for severe mental disorders, it is important to address mental health issues in school settings and promote prevention and early intervention programs.

**Disclosure of Interest:**

None Declared

